# Structural Dynamics of P-Rex1 Complexed with Natural Leads Establishes the Protein as an Attractive Target for Therapeutics to Suppress Cancer Metastasis

**DOI:** 10.1155/2023/3882081

**Published:** 2023-12-07

**Authors:** Alaa R. Hameed, Sama Fakhri Ali, Sarah M. S. Alsallameh, Ziyad Tariq Muhseen, Nahlah Makki Almansour, Naif ALSuhaymi, Mahdi H. Alsugoor, Khaled S. Allemailem

**Affiliations:** ^1^Department of Medical Laboratory Techniques, School of Life Sciences, Dijlah University College, Baghdad, Iraq; ^2^Department of Anesthesia Techniques, School of Life Sciences, Dijlah University College, Baghdad, Iraq; ^3^Ministry of Higher Education and Scientific Research, Gilgamesh Ahliya University College, College of Health and Medical Techniques, Department of Medical Laboratories Techniques, Baghdad, Iraq; ^4^Department of Pharmacy, Al-Mustaqbal University College, Hillah, Babylon 51001, Iraq; ^5^Department of Biology, College of Science, University of Hafr Al Batin, Hafr Al Batin 31991, Saudi Arabia; ^6^Department of Emergency Medical Services, Faculty of Health Sciences, AlQunfudah, Umm Al-Qura University, Mecca 21912, Saudi Arabia; ^7^Department of Medical Laboratories, College of Applied Medical Sciences, Qassim University, Buraydah 51452, Saudi Arabia

## Abstract

Phosphatidylinositol 3,4,5-trisphosphate- (PIP3-) dependent Rac exchanger 1 (P-Rex1) functions as Rho guanine nucleotide exchange factor and is activated by synergistic activity of G*βγ* and PIP3 of the heterotrimeric G protein. P-Rex1 activates Rac GTPases for regulating cell invasion and migration and promotes metastasis in several human cancers including breast, prostate, and skin cancer. The protein is a promising therapeutic target because of its multifunction roles in human cancers. Herein, the present study attempts to identify selective P-Rex1 natural inhibitors by targeting PIP3-binding pocket using large-size multiple natural molecule libraries. Each library was filtered subsequently in FAF-Drugs4 based on Lipinski's rule of five (RO5), toxicity, and filter pan assay interference compounds (PAINS). The output hits were virtually screened at the PIP3-binding pocket through PyRx AutoDock Vina and cross-checked by GOLD. The best binders at the PIP3-binding pocket were prioritized using a comparative analysis of the docking scores. Top-ranked two compounds with high GOLD fitness score (>80) and lowest AutoDock binding energy (< -12.7 kcal/mol) were complexed and deciphered for molecular dynamics along with control-P-Rex1 complex to validate compound binding conformation and disclosed binding interaction pattern. Both the systems were seen in good equilibrium, and along the simulation time, the compounds are in strong contact with the P-Rex1 PIP3-binding site. Hydrogen bonding analysis towards simulation end identified the formation of 16 and 22 short- and long-distance hydrogen bonds with different percent of occupancy to the PIP3 residues for compound I and compound 2, respectively. Radial distribution function (RDF) analysis of the key hydrogen bonds between the compound and the PIP3 residues demonstrated a strong affinity of the compounds to the mentioned PIP3 pocket. Additionally, MMGB/PBSA energies were performed that confirmed the dominance of Van der Waals energy in complex formation along with favorable contribution from hydrogen bonding. These findings were also cross-validated by a more robust WaterSwap binding energy predictor, and the results are in good agreement with a strong binding affinity of the compounds for the protein. Lastly, the key contribution of residues in interaction with the compounds was understood by binding free energy decomposition and alanine scanning methods. In short, the results of this study suggest that P-Rex1 is a good druggable target to suppress cancer metastasis; therefore, the screened druglike molecules of this study need *in vitro* and *in vivo* anti-P-Rex1 validation and may serve as potent leads to fight cancer.

## 1. Introduction

Metastasis is one of the hallmark features of various cancer types characterized by the circulating tumors which spread all across the body affecting the physiology of distant organs [1]. Cancer is, therefore, a fatal disease due to its metastatic nature, and barely a few of the anticancer agents to date have been able to reduce tumor growth [2–5]. Interestingly, with the discovery of small molecules, a new anticancer therapy has been introduced that can potentially inhibit different enzymes with a vital role in cancerous tumor development and metastasis [6–8].

Among notable markers is PIP3-dependent Rac, an important regulator in the metastasis of various cancer types such as prostate cancer, melanoma, and breast cancer [9, 10]. Further reports of experimentally used lab cell lines suggested that the P-Rex enzymes are attributive to invasive and migration phenotypes [11]. Henceforth, both P-Rex enzyme isoforms (P-Rex1 and P-Rex2) are significant therapeutic targets as their overexpression leads to a clinical manifestation of cancer metastasis. Previous reports suggested that the mouse model remained viable and healthy and has only shown mild symptoms of neutrophilia, suggesting very minimum adverse drug reaction with the loss of function of P-Rex1 [12]. Moreover, the dRouble knockdown model has shown a decline in melanoblast, migration, and, hence, metastatic resistance [10]. Hence, the molecular insight into the regulatory pathway involving P-Rex1 activity could be better understood which could lead to the discovery of anticancer (metastatic drugs) therapeutics in the future. Dbl Rho guanine nucleotide exchange factors (RhoGefs) are a subfamily of P-Rex. The major characteristics include the presence of P-Rex2/2b isoforms [13] and tandem Dbl homology (Dh)/pleckstrin homology (pH) domains [14]. Moreover, both P-Rex1 and P-Rex2 share the homology to inositol polyphosphate-4-phosphatase (IP4P), marked by the presence of a C-terminal domain, N-terminal DH/pH domains, two Dep domains, and two Pdz domains. However, both enzymes lack phosphatase activity [15].

Whilst P-Rex1 is highly expressed by the brain and neutrophils [14], a second isoform, P-Rex2 I is strongly expressed in the skeletal muscles, small intestine, and placenta [16]. Moreover, PIP3 has been involved in synergistic and direct activation of G protein BG subunits and all isoforms [17]. These isoforms are selective for Cdc42 and Rac-subfamily (Rho GTPases), and P-Rex can, therefore, participate in the signaling pathway of both receptors (tyrosine kinases). This, in turn, activates phosphoinositide 3-kinase (PI3K) to facilitate the increase in PIP3 expression, together with G protein-coupled receptors (GPCRs), and GbG subunits are then released. For instance, changes in the actin cytoskeleton are mediated by the activation of P-Rex1 which is stimulated by platelet-derived growth factor receptor [14] and by the formyl peptide receptors (chemotactic) found in neutrophils [18]. The extent of activation and, hence, the levels of P-Rex1 are monitored in two ways: via its recruitment to the cell membrane—a site where PIP3 and GbG subunits are produced, with its GTPase substrate, and secondly, by measuring the acceleration of P-Rex1-mediated nucleotide exchange on Cdc42, Rac1, and Rac2 [19].

Nevertheless, the mechanisms by which P-Rex1 is regulated involving these participating molecules or domains (outside its catalytic core) are still not fully understood. Through truncation studies, it was revealed that the higher activity is demonstrated by the short N-terminal P-Rex1 dh/ph fragment than the full-length counterpart. This explains the autoinhibition of enzyme-mediated by the C-terminal domains [12], with the PH domain of the protein (P-Rex1) being the significant player in the stimulation of PIP3 [6]. On the contrary, the location of the GbG-binding site is still questionable as per many reports [20].

**Figure 1:**
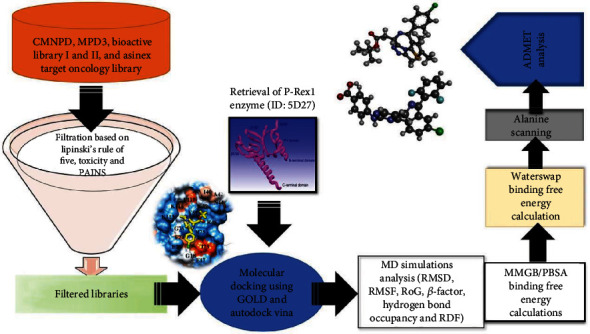
Schematic presentation of the complete framework of study highlighting step-by-step approach to identify hit compounds against P-Rex1 enzyme.

Inspired by the reports suggesting the therapeutic potential of the P-Rex1 enzyme [6], and the need for the development of new small molecule inhibitors to control cancer metastasis, the current study was conducted. The study was commenced by applying Lipinski's rule of five (RO5) [21], toxicity [22], and filter pan assay interference compounds (PAINS) [23] filters on diverse compound libraries collected from natural as well as synthetic sources. After, structure-based virtual screening (SBVS) [24] was conducted to filter the libraries against the P-Rex1 enzyme to disclose the best binding molecules with a rich pattern of chemical interactions at an atomic level. The filtered hit potency was subsequently validated by several state-of-the-art molecular dynamics (MD) simulation-based [25–27] analyses including prominent molecular mechanics generalized Born surface area (MM/GBSA) and molecular mechanics Poisson-Boltzmann surface area (MM/PBSA) methods [26, 28, 29] as well as by more sophisticated WaterSwap absolute binding free energy method [30, 31]. The enzyme hotspot residue involved in major interaction energy contribution then underwent site-directed mutagenesis to uncover their role in compound binding [32, 33]. Computational pharmacokinetics was done to ensure selection of molecules with higher chances to reach the marked [34–36]. The lead compounds identified in this study might be subjected to *in vivo* and *in vitro* biological validation assays to determine the molecule's actual anti-P-Rex1 enzyme activity. A schematic presentation of steps followed in this study to identify potential P-Rex1 inhibitors is illustrated in Figure 1.

## 2. Materials and Methods

### 2.1. Inhibitor Library and P-Rex1 Enzyme Preparation

Several different libraries including comprehensive marine natural product database (CMNPD) [37], medicinal plant database for drug designing (MPD3) [38], selleckchem bioactive library I (https://www.selleckchem.com/screening/chemical-library.html), selleckchem bioactive library II (https://www.selleckchem.com/screening/bioactive-compound-library-2.html), and Asinex target oncology library (http://www.asinex.com/oncology-targeted-oncology/) were used in the screening process. Compounds of the libraries were derived from natural sources as well as contained synthetic ready-to-use molecules. Prior to their use in structural-based virtual screening (SBVS) [39], the libraries were filtered based on Lipinski's rule of five (RO5) [21], toxicity, and filter pan assay interference compounds (PAINS) [23]. The hydrogen bonds and charges were added to the ligands in UCSF Chimera 1.15 [40] to prepare them for docking. These steps were followed by the energy minimization via steepest descent and conjugate gradient algorithms [41]. The P-Rex1 enzyme was retrieved from Protein Data Bank (PDB) (ID: 5D27, organism: Homo sapiens) [6], the enzyme structurally is a monomer with an overall structure resolution of 1.92 Å (https://www.rcsb.org/structure/5d27). The enzyme was then treated in UCSF Chimera 1.15 to discard water molecules and extra ligands that are not functionally relevant. Afterward, an energy minimization run was performed to remove steric clashes if found any. The minimized and unminimized enzymes were compared and contrasted via the Ramachandran plot [42], and the best structure based on a high number of residues plotting in Rama favored and less number of residues in disallowed regions was selected. The optimal enzyme structure was used as receptor molecule in the virtual screening of the libraries discussed above.

### 2.2. Comparative Docking Studies

To assess binding affinity of library compounds with the P-Rex1 enzyme, we used genetic optimization for ligand docking (GOLD) 5.2 [43] and PyRx AutoDock Vina [44] software. In GOLD, 100 iterations for each library compound were considered while keeping parameters like island number, population size, niche size, and number of generic operations as default. The hydrogen bond and Van der Waals cut-off distance set of 4.0 Å and 2.5 Å, respectively, were employed to ensure the selection of only those complexes having short-distance intermolecular interactions. In AutoDock Vina, binding mode iterations were set to 100, and the maximum energy difference to 3 kcal/mol. Best hit shortlisting was based on the good binding energy profiles: high GOLD fitness score and AutoDock lowest binding energy. In both docking softwares, site-directed docking of the compounds was performed where PH domain of the P-Rex1 Enzyme was targeted. The PH domain is the binding site of PIP_3_ and can be considered for rational design of small molecule inhibitors [6]. The PIP_3_ was used as a control. The PIP3 is a natural and high-affinity binder of the P-Rex1 PH domain, and its competitive blockage can stop the natural function of P-Rex1 [45].

### 2.3. Molecular Dynamics (MD) Simulations

MD simulation studies were performed to study the physical dynamics of atoms and molecules in protein-ligand docked complex [25, 46, 47]. The top 2 hits were chosen, and MD simulations with a run time of 100 ns with each compound were conducted. We used AMBER20 [48, 49] for MD simulations where input ligand and receptor files for LEaP [50] were prepared automatically. Next, the P-Rex1 enzyme-compound complexes underwent hydrogen bond addition, water molecule removal, and assigning bond order; any missing sidechains were filled, and pH was adjusted to 7. The enzyme was treated with FF14SB [51] while compound parameterization was done through AMBER general force field (GAFF) [52]. The simulation system was kept solvated in a TIP3P water box (as an example, top-1 compound complex with P-Rex1 is shown in S-Figure 1). The boundaries that had dimensions of the box were $10\;\overset{{}^{\circ}}{\text{A}}\times 10\;\overset{{}^{\circ}}{\text{A}}\times 10\;\overset{{}^{\circ}}{\text{A}}$ around the docked complex. The systems were kept neutralized involving the addition of CL− or NA+ counter ions. Energy minimization was performed by both steepest descent and conjugate gradient methods for 1500 rounds [32]. The systems were heated with a gradual rise of temperature to 300 K, followed by equilibration for 100 ps at a constant 300 K temperature [41]. MD simulation production run was accomplished for 100 ns with NPT (constant number of particles, pressure (1.01 bar), and temperature (300 k)) employing the smooth particle mesh Ewald (PME) method [53] to treat long-range interactions. SHAKE [54] and the Langevin thermostat [55] were applied to constrain atoms involved in covalent interactions with hydrogen atoms and keep temperature constant, respectively. Subsequently, CPPTRAJ [56] was utilized to analyze the trajectories for the parameters including root mean square fluctuation (RMSF) [57], root mean square deviation (RMSD) [58], radius of gyration (RoG) [59], beta factor (*β*-factor) [60], hydrogen bonding [61], and radial distribution function (RDF) [62]. RDF describes probability of finding particles with respect to a reference particle at distance “r” [62]. The RDF plotting is commonly applied post MD simulations to highlight key residue atom density distribution with ligand with respect to distance [63–65].

### 2.4. MMGB/PBSA Binding Free Energy Calculations

For the binding energy estimation, all simulation trajectories were analyzed via MM/GBSA and MM/PBSA techniques. The recording interval was adjusted to 1000 ps for entire 100 ns simulation. We gathered 100 frames to calculate MMGB/PBSA through the MM/PBSA.py module [66] of AMBER20.

The following mathematical equation was used for calculating the binding free energies:

(1) $$\begin{array}{c} \Delta G_{\text{BINDING}}=\Delta G_{\text{COMPLEX}}-\left[\left(\Delta G_{\text{Compound}}\right)+\Delta G\;\left.\text{P}-\text{Rex}1\right)\right], \end{array}$$

and

(2) $$\begin{array}{c} \Delta G_{\text{BINDING}}=\Delta G_{\text{MM}}+\Delta G_{\text{PB}}+\Delta \text{GSA}-T\Delta S, \end{array}$$

where ΔTDS is conformation entropic contribution, ΔGMM is molecular mechanics' energy including Van der Waals electrostatic interaction, and ΔGSA considers both polar solvation energy and nonpolar solvation energy [32]. The entropy energy was estimated using a bash script by Duan et al. based on the simulation trajectories [67].

### 2.5. Binding Free Energy Calculation WaterSwap

Additionally, cross-validation of the binding free energy estimation was performed using WaterSwap following the protocol described by Woods et al. [30].

### 2.6. Alanine Scanning

Alanine scanning experiment performed net to mutate key residues of the enzyme involved in close distance interactions with the compounds and is important for stable binding of the compounds at the docked site [68]. Mutants are created manually by replacing the enzyme residues (Lys39 and Arg75) with ALA and then loading the enzyme structure into AMBER LEaP. The simulation was again performed with mutated enzyme structure, and MMGB/PBSA energy was reestimated. The difference in the binding free energy between the native and mutant P-Rex1 enzyme was estimated using the below equation.

(3) $$\begin{array}{c} \Delta \Delta G_{\text{binding}}=\Delta G_{\text{binding}}\text{ of wild type}-\Delta G_{\text{binding}}\text{ of mutant}. \end{array}$$

A higher value of Δ*G*_binding_ of mutant specifies that the mutant is less stable compared to the wild type. Accordingly, positive ΔΔG_bind_ value demonstrates favorable contribution and vice versa [32].

### 2.7. ADMET Analysis

The shortlisted top-2 compounds were selected to study their ADMET properties using SWISSADME [35], pkCSM [34], and PreADMET [69]. These softwares assessed *in vivo* absorption parameters such as water solubility, gastrointestinal absorption, *in vivo* caco2 cell permeability, and p-glycoprotein inhibition. Additionally, different cytochrome p450-type inhibitions were also evaluated for the compounds. For assessing the distribution property such as central nervous system (CNS) permeability, Lipinski's rule (rule of five) [21], and blood-brain barrier (BBB) [70] penetration, the toxicity assessment was conducted using a range of endpoints such as AMES test [71], carcinogenicity test in mouse, and rat acute algae toxicity. Measuring the level of excretion is also one of the major drug clearance tests, as many over-the-counter drugs are removed from the market shelf due to their poor renal clearance profile [72]. In our present study, parameters like total renal clearance as well as renal oct2 substrate to predict the extent of excretion efficiency of the proposed compounds were included.

## 3. Results and Discussion

### 3.1. Identification of Potential P-Rex1 Enzyme Inhibitors

Virtual screening of the libraries discussed in the methodology was performed against P-Rex1 enzyme PIP3-binding pocket through GOLD docking software, and compound ranking was done as per GOLD fitness score. The higher the score, the better is docking affinity of the compound for the enzyme [41]. The top 50 best hits were shortlisted and redocked to the enzyme using AutoDock Vina in PyRx to reaffirm its binding potential. Comparative scoring function analysis was then performed, and Top-2 hits were identified. The 3D structure of these two compounds along with scoring functions is tabulated in Table 1.

**Figure 2:**
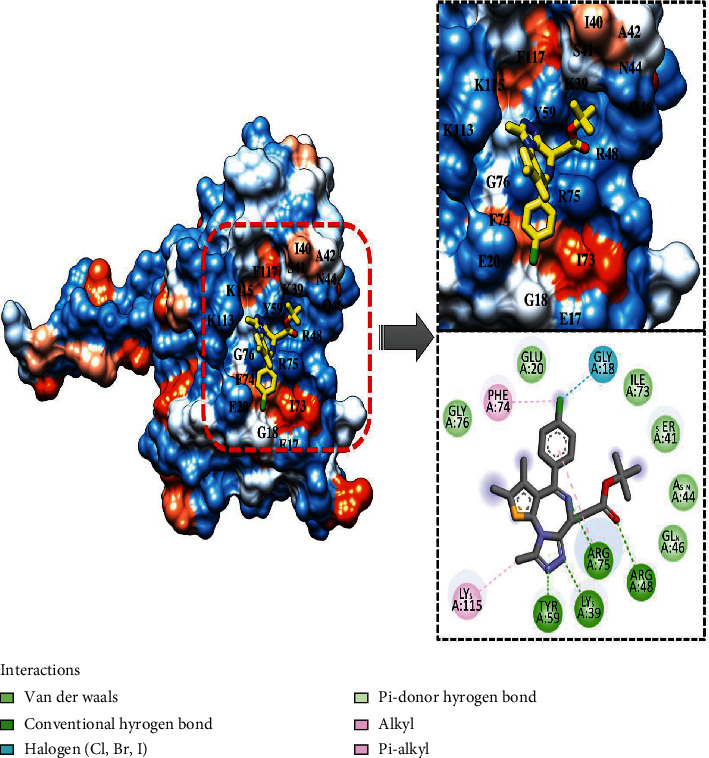
Docked top-1 compound at the PIP_3_ active pocket, close view of its interacting residues, and presentation of different types of chemical bonding.

For comparative analysis, a control PIP_3_ was run to assess the inhibitory potential of the compounds. The GOLD fitness score of top-1 compound (O=C(OC(C)(C)C)CC1N=C(c2ccc(cc2)Cl)c2c(n3c1nnc3C)sc(c2C)C) is 82.5, and its binding energy value is -12.8 kcal/mol. For top-2 (OC(=O)C1=CC=C(NC2=NC3=C(CN=C(C4=C3C=CC(=C4)Cl)C5=C(F)C=CC=C5F)C=N2)C=C1), the GOLD fitness score and binding energy are 82.1 and -12.6 kcal/mol, respectively. Both the compounds produced a rich pattern of chemical interactions with residues of the active pocket. For example, top-1 compound formed hydrogen bonds with Lys39, Arg48, Tyr59, and Arg75 and Van der Waals interactions with Glu20, Ser41, Asn44, Gln46, and Gly76. The compound is also engaged via alkyl interaction by Phe74 and Lys115 (Figure 2).

**Figure 3:**
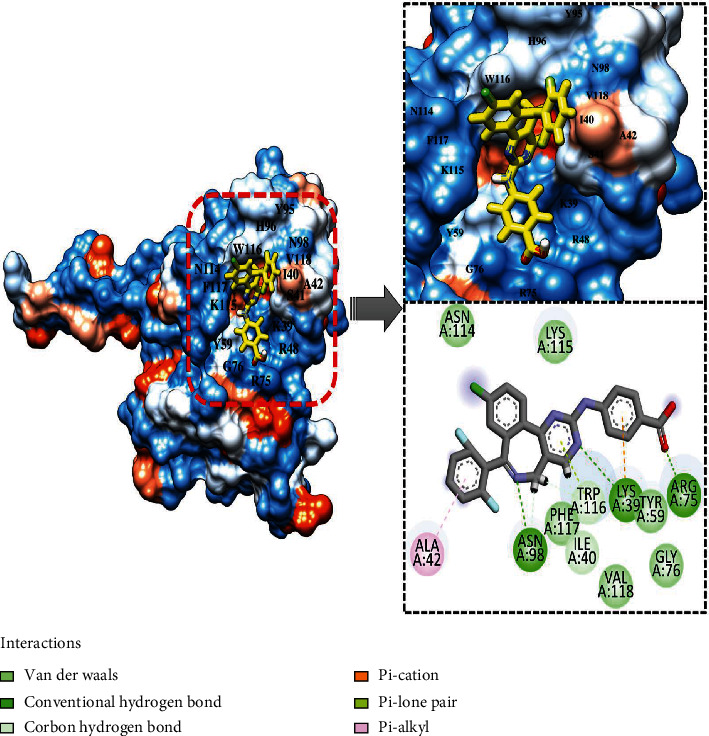
Docked top-2 compound at PIP_3_ active pocket, close view of its interacting residues, and presentation of different types of chemical bonding.

On the other hand, the top-2 compound generates a hydrogen bond network with Lys39, Arg75, and Asn98. Van der Waals interactions involve residues Ile40, Tyr59, Gly76, Asn114, Lys115, Trp116, Asn117, and Val118. Only one residue Ala42 was reported in Pi-alkyl interaction (Figure 3). In general, the binding conformation of both compounds was as such to access the floor of the PIP_3_ active pocket and accommodate themselves along the length. This is why the majority of the enzyme residues involved in interactions were found the same in compound docking. It was observed that small chemical moieties of the compounds adjusted well inside the pocket while larger chemical structures protrude outside. This further hints that the compounds following Lipinski's rule of five could be better led in the future for structure as well as biological activity optimization.

### 3.2. MD Simulations of Complexes

**Figure 4:**
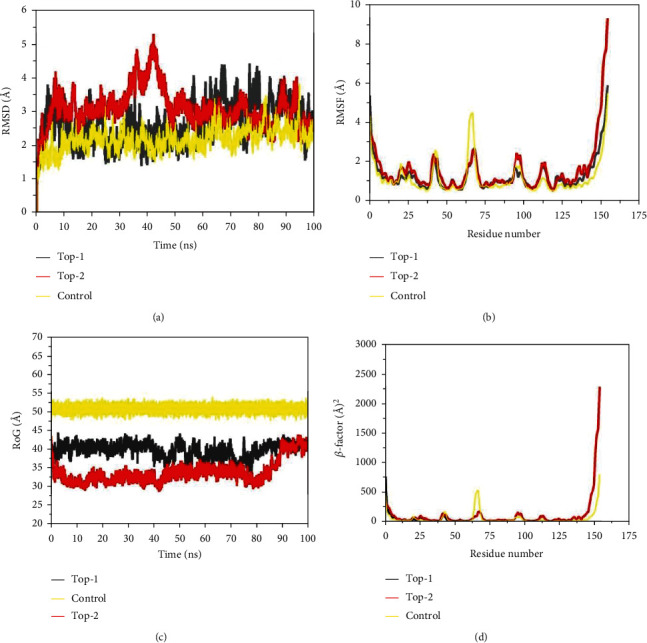
Different statistical parameters were calculated for simulated complexes. (a) RMSD, (b) RMSF, (c) RoG, and (d) *β*-factor.

Molecular docking is a powerful approach in predicting ligand molecule binding mode and interactions with respect to a receptor macromolecule, yet the docked intermolecular conformation of molecules is static, and hence, a full description of the dynamic stability of the selected complexes must be evaluated in a biological environment [73, 74]. MD simulation in this regard is a useful technique to mimic complex physical motion in actual environment and interpret intermolecular affinity and overall stability of compound interactions with the P-Rex1 enzyme active site residues [25, 65]. RMSD, which is a significant parameter in determining complex dynamic stability and overall equilibrium, was calculated first as shown in Figure 4(a) [57, 58, 75]. The mean carbon alpha RMSD of the systems are in the following order: top-1 ($2.66\;\overset{{}^{\circ}}{\text{A}}\pm 0.58$), top-2 ($3.09\;\overset{{}^{\circ}}{\text{A}}\pm 0.56$), and control ($2.19\;\overset{{}^{\circ}}{\text{A}}\pm 0.33$). These RMSD values are demonstrating highly acceptable range of system equilibrium. Because of the small size of the enzyme, the N- and C-terminals suffer from fluctuations, thus pushing the overall structure more dynamics; however, because of the greater strength of the docked compounds to the enzyme, the compound conformation at the docked pocket is not altered and remained in stable pose throughout the length of simulation time. A bit higher fluctuation in top-2 compound RMSD was noticed around 30 ns to 50 ns, which, upon inspection, is due to flexible dynamics of the enzyme loops, but it does not alter the compound binding at all. To further validate complex stability, residue level RMSD, more technically called RMSF, was measured (Figure 4(b)) [76, 77]. The mean carbon alpha RMSD for the systems is top-1 ($1.29\;\overset{{}^{\circ}}{\text{A}}\pm 0.83$), top-2 ($1.55\;\overset{{}^{\circ}}{\text{A}}\pm 1.27$), and control ($1.17\;\overset{{}^{\circ}}{\text{A}}\pm 0.87$). As stated earlier, the C- and N-terminal residues of the enzyme are highly unstable in dynamic environment which contributes to its high RMSF, but it does not alter compound binding and interactions. Next, RoG analysis [59] was performed to understand the 3D compactness of the P-Rex1 enzyme in the presence of the compounds and control (Figure 4(c)). The mean carbon alpha RoG for top-1, top-2, and control are $44.56\;\overset{{}^{\circ}}{\text{A}}\pm 39.88$, $33.96\;\overset{{}^{\circ}}{\text{A}}\pm 2.97$, and $50.95\;\overset{{}^{\circ}}{\text{A}}\pm 0.96$, respectively. The RoG analysis agrees on good compactness of the enzyme, and no significant structural deviations in the enzyme structure were detected in the presence of the compounds. Thus, it can be inferred that the enzyme is enjoying the company of compounds and has well accommodated the compounds in its pocket. The RoG findings are in line with the RMSD analysis of the complexes. Lastly, the *β*-factor [57] parameter for the complexes was investigated that calculated the mean *β*-factor as follows: top-1 ($56.31\;{\overset{{}^{\circ}}{\text{A}}}^{2}\pm 106.0$), top-2 ($106.68\;{\overset{{}^{\circ}}{\text{A}}}^{2}\pm 272.71$), and control ($56.25\;{\overset{{}^{\circ}}{\text{A}}}^{2}\pm 110.81$). The *β*-factor plots are depicting the same behavior of the complexes like that of RMSF and are analogous (Figures 4(b) and 4(d)).

### 3.3. Hydrogen Bond Occupancy Analysis

Hydrogen bonds are central to determine ligand binding specificity and valuable chemical interactions to enhance the strength of receptor-ligand binding [61]. Hydrogen bond occupancy was determined to shed light on the role of residues involved in compound bindings via hydrogen bonds. For top-1 and top-2 compounds, 16 and 22 hydrogen bonds were detected with the P-Rex1 enzyme through different percentages of occupancy as can be seen in Table 2. This increased number of hydrogen bonds between the enzyme and the compounds strongly suggests their vital role in the stability of complexes.

### 3.4. High-Density Intermolecular Interaction RDF Plotting

**Figure 5:**
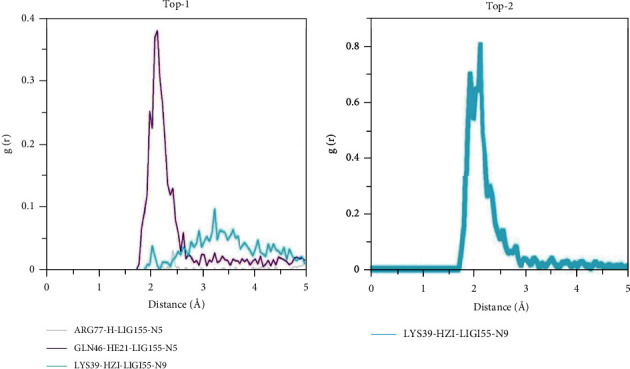
RDF plots for some key interactions keeping the compounds intact at the docked site.

For the top-1 compound, three key residues that are also reported by docking studies were used in RDF plotting throughout the length of MD simulations. These interactions include that residues Lys39, Gln46, and Arg77 have engaged compound N5 and N7 atoms for holding the ligand at the docked pocket. Among the interactions, Gln46 residue interaction via its HE21 atom to engage top-1 compound N5 atom in hydrogen bonding has the high interatomic density distribution. The maximum g(r) value noticed for this interaction is at 2.1 Å with g(r) value of 0.37. The other two interactions presented in the figure are more dispersed and have varied intermolecular density distribution at different distance points. For the top-2 compound, only one, i.e., Lys39 HZ1, atom that contacted the compound through the N9 atom is more refined and played a significant contribution in ligand stability. The maximum g(r) value of this interaction is 0.8 at a distance of 2.12 Å. RDF plots of top-1 and top-2 compounds are shown in Figure 5.

### 3.5. Estimation of MM/GBSA and MM/PBSA Binding Free Energies

The MM/GBSA and MM/PBSA methods are significantly better choices than conventional docking scoring functions in order to evaluate the binding affinity of compounds to the target biological macromolecule as well as in terms of examining conformation ordering performance [28, 78]. Both these methods used simulation trajectories to generate atomic-level different chemical interaction energies in gas and solvation phase. Overall, the binding free energies estimated for the systems are presented in Table 3. The complexes depicted good intermolecular stability with the net binding energy of -30.06 kcal/mol, -14.35 kcal/mol, and -20.51 kca/mol for top-1, top-2, and control, respectively, in MM/GBSA. Hence, in MM/GBSA, the top-1 compound complex with P-Rex1 enzyme is more stable compared to top-2 and control; though both the complexes have high stability. In the case of MM/PBSA, the systems scored less net binding energy value because of the high solvation energy where polar energy contributed unfavorably to the complex stability. The control system has deciphered more energy stable than the compound complex. Both MM/GBSA and MM/PBSA methods concluded the favorable nature of Van der Waals energy in compounds/control interaction with the targeted enzyme while negative contribution was reported from electrostatic and polar solvation energies. A discrepancy was noted between the docking scores and MMGB/PBSA methods, as the former is less accurate than the latter. The difference between MM/GBSA and MM/PBSA net value is that the former uses a generalized approximation of the Poisson-Boltzmann equation and is faster treatment than that of MM/PBSA. The difference is also due to the polar solvation effect [32]. Additionally, entropy analysis was performed and revealed that the entropy energy of top-1 is 4 kcal/mol and top-2 is 1 kcal/mol.

### 3.6. WaterSwap Energy Calculation

**Figure 6:**
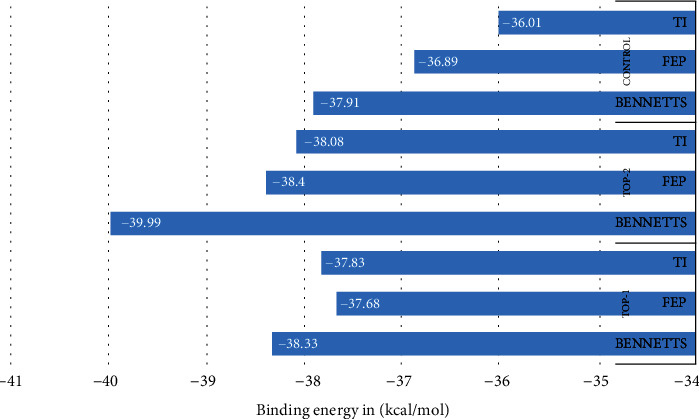
WaterSwap binding free energies calculated for the complexes by three different methods.

The MM/GBSA and MM/PBSA binding free energy calculation suffers from several drawbacks and therefore requires additional validation by another more sophisticated approach that calculates absolute binding free energies like WaterSwap [30]. The method applies the construction of a reaction coordinate which allows swapping of bounded ligand to the protein with an equivalent size of water molecules. The MM/GBSA and MM/PBSA use implicit water models that skip details about ligand water and protein-water interactions which is of high importance as some water molecules may form bridging interactions between the ligand and its receptor macromolecule. Also, these methods do not include entropy energy which must be taken into account while calculating binding free energies. The WaterSwap calculates absolute binding free energy via three principles, i.e., Bennetts, free energy perturbation (FEP), and thermodynamic integration (TI) [30]. As presented in Figure 6, all the three complexes are highly stable by scoring very less in the WaterSwap calculation. The system WaterSwap calculations are well converged as the differences among the energy values are <1 kcal/mol, suggesting good overall stability and intermolecular affinity of the complexes [64].

### 3.7. Decomposition of MM/GBSA Binding Free Energy

**Figure 7:**
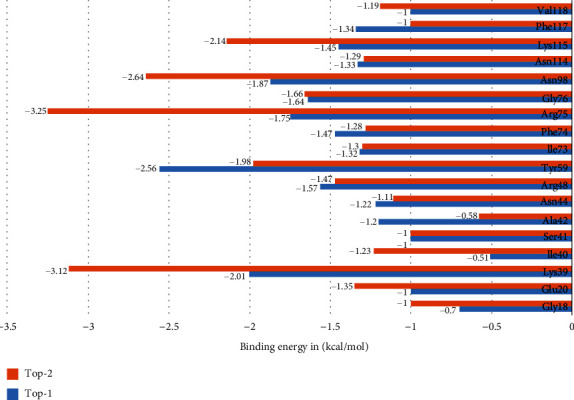
Net MM/GBSA binding free energy decomposition into residues that dominated interactions with the compounds.

Atomic-level understanding of P-Rex1-compound interactions was achieved by decomposing the net MM/GBSA binding energy into residues that are present within or around the enzyme active pocket. Residues that contributed majorly to the interaction by securing <−1 kcal/mol are termed as hotspot residues and plotted in Figure 7. These residues include Gly18, Glu20, Lys39, Ile40, Ser41, Ala42, Asn44, Arg48, Tyr59, Ile73, Phe74, Arg75, Gly76, Asn98, Asn114, Lys115, Phe117, and Val118. All these residues are seen to form close distance hydrophobic and hydrophilic interactions and are part of the active pocket. As observed in the net binding energy of the systems, Van der Waals energy dominates the overall interaction of these residues with the compounds whereas nonfavorable contribution from electrostatic and polar solvation energy was also revealed.

### 3.8. Alanine Scanning Analysis

The key contribution of selected hotspot residues to compound binding was understood by mutating the residues to alanine. Considering the major contribution of Lys39, and Arg75 in both compound binding and stable docking, site-directed mutagenesis was performed. Following mutating residues, molecular dynamics simulation was performed of the same length initially performed, and residue-wise MM/GBSA binding free energy was recalculated. It was reported the mutated residues steered a decline in binding energy, thus owing to the importance of both residues towards enzyme functionality and compound binding. The binding energy of Lys39 and Arg75 was seen reduced to 0.45 kcal/mol and -1.0 kcal/mol from the original -2.01 kcal/mol and -1.75 kcal/mol, respectively.

### 3.9. Computational Druglikeness and Pharmacokinetics

In-depth details of druglikeness and pharmacokinetics of the compounds are tabulated in Table 4. From druglikeness perspectives, both the compounds fulfill prominent Lipinski's rule of five. However, in addition to Lipinski, top-1 also completely covers Ghose et al. [79], Veber et al. [80], Egan et al. [81], and Muegge et al. [82] druglike rules. The top-2 compound also accepts the Veber rule but not others. The druglike analysis of the compounds suggests that the compounds could be good candidates for further optimization and have a good chance to reach the market. The lipophilicity Log*P* value of the compounds is within the acceptable range, thus indicating its positive impact on drug metabolism and uptake [80]. The good Log*P* also makes sure that the compounds do not bind to off-target and unwanted cellular targets. The topological surface area (TPSA) score of the compounds is also within the limit of good druglike molecules, therefore, increasing the chances of the compounds to penetrate the cells and increasing compound absorption [80]. High gastrointestinal (GI) absorption is key in novel drug design as this guarantees that a high concentration of drug reaches the target site and performs the required therapeutic action [83]. Both the selected compounds have high GI absorption and could be potential oral candidates. The compounds can not cross the blood-brain barrier (BBB) and are nonsubstrates of P-glycoprotein (P-gp) [84] v. The nonsubstrate nature of the compounds will allow the compounds to keep a good concentration of the drugs in blood plasma and will not affect their final therapeutic effects. From a medicinal chemistry point of view, both compounds are easy to synthesize and do not contain any pan assay interference compounds (PAINS) alerts [23]. The zero alert for PAINS means that the compounds interact only with P-Rex1 and are thus selective in nature. The top-1 compound is negative for all toxicity tests whereas top-2 is only positive for hepatotoxicity. Lastly, both compounds were predicted not to serve as substrates for organic cation transporter 1 (OCT2); therefore, the compounds may deposit and do not undergo renal clearence [34].

## 4. Conclusions and Future Prospects

The present study explored several druglike, nontoxic, and PAINS alert-free libraries against the P-Rex1 enzyme to block cancer metastasis. The findings provided a detailed information about two shortlisted inhibitory molecules: ((O=C(OC(C)(C)C)CC1N=C(c2ccc(cc2)Cl)c2c(n3c1nnc3C)sc(c2C)C and OC(=O)C1=CC=C(NC2=NC3=C(CN=C(C4=C3C=CC(=C4)Cl)C5=C(F)C=CC=C5F)C=N2)C=C1)) that showed descent affinity for the enzyme as validated by range of computational analysis. The compounds docked efficiently at the PIP3-binding pocket and were dominated by a mixture of Van der Waals and hydrogen bonding. The static docked complexes of the compounds were subjected to MD simulation dynamics, and structural stability was affirmed by several different statistical parameters such as RMSD, RMSF, RoG, *β*-factor, hydrogen bond occupancy, and RDF. Moreover, MM/GBSA, MM/PBSA, and WaterSwap methods were employed to examine the predictions made by docking and MD simulation studies that agree to the findings and categorized the complexes as stable. Alanine scanning was also performed to induce site-directed mutagenesis of Lys39 and Arg75 to alanine and evaluate its contribution in compound binding. The analysis confirmed the said residues to play a significant role in the overall stability of the compounds at the docked site. In-depth ADMET studies of the compounds showed the molecules to have excellent druglikeness, pharmacokinetics, medicinal chemistry, and nontoxic profiles. Previously, six small molecules were identified, which interact with the same P-Rex1 PH domain reported herein. Out of six, three of the compounds inhibit N-formylmethionyl-leucyl-phenylalanine of the enzyme and block neutrophils spreading along with GTPase Rac2, thus affecting downward P-Rex1 activity [45]. The scope of the study was to computationally identify inhibitory molecules of the P-Rex1 enzyme to stop the regulation of cell invasion and migration and promote metastasis in several human cancers including breast, prostate, and skin cancer. Such studies have been regularly applied in the last decades, and many molecules have been unveiled computationally and validated experimentally to show biological potency. The study is open for experimentalists to use the compounds in biological *in vivo* and *in vitro* assays to disclose their real biological potential. The outcomes of the study will not only speed the discovery of the P-Rex1 enzyme but also deliver ready-to-use data for specific experimental testing.

## Figures and Tables

**Table 1:** GOLD fitness score and binding energy of the two hits and control.

Hit	Compound 3D structure	Gold fitness score	AutoDock Vina binding energy in kcal/mol
Top-1	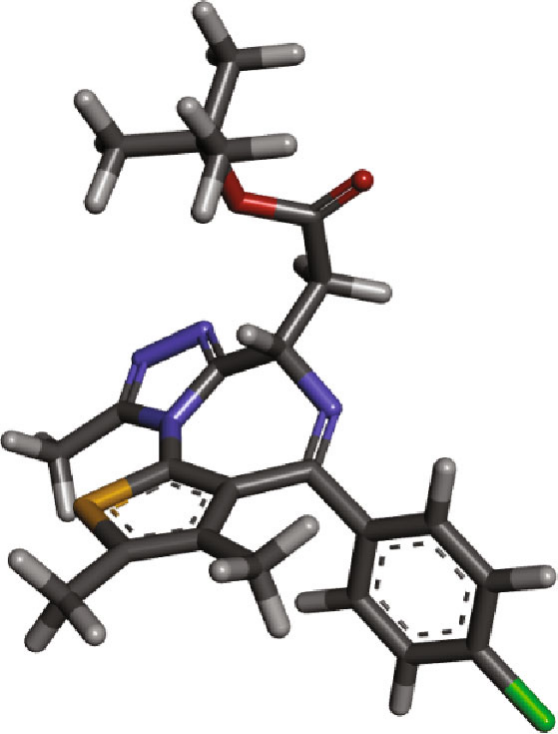	82.5	-12.8

Top-2	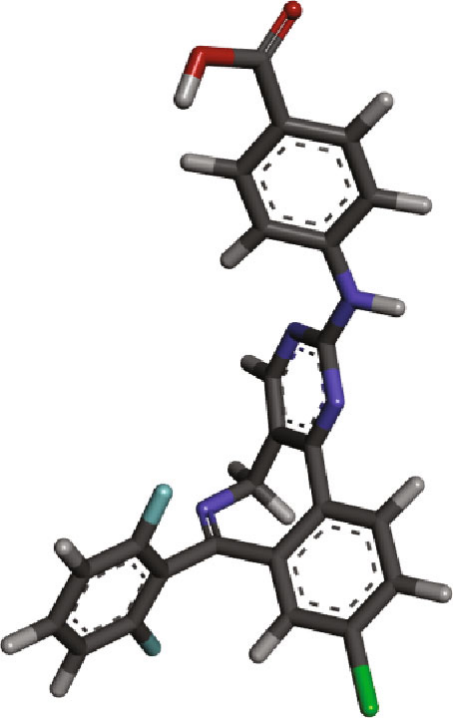	82.1	-12.6

Control	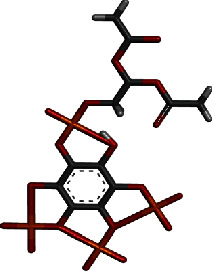	75.14	-11.7

**Table 2:** Hydrogen occupancy analysis for top-1 and top-2 compounds based on MD simulation trajectories.

Donor	Acceptor	Occupancy (%)
*Top-1*		
ARG75-Side-NH2	LIG155-Side-N9	0.07
ARG77-Main-N	LIG155-Side-N5	0.23
ARG77-Main-N	LIG155-Side-N1	1.14
TYR59-Side-OH	LIG155-Side-O27	0.05
LYS113-Side-NZ	LIG155-Side-O27	0.02
TYR59-Side-OH	LIG155-Side-O26	0.01
ASN110-Side-ND2	LIG155-Side-O27	0.49
SER41-Side-OG	LIG155-Side-N5	0.50
LYS39-Side-NZ	LIG155-Side-O27	0.01
LYS39-Side-NZ	LIG155-Side-O26	0.01
GLN46-Side-NE2	LIG155-Side-N5	0.45
GLN46-Side-NE2	LIG155-Side-N1	0.09
LYS39-Side-NZ	LIG155-Side-N9	0.01
SER41-Side-OG	LIG155-Side-N1	0.08
LYS39-Side-NZ	LIG155-Side-N5	0.04
GLN46-Side-NE2	LIG155-Side-O27	0.02

*Top-2*		
ASN98-Side-ND2	LIG155-Side-N22	0.28
LIG155-Side-N25	ASN98-Side-OD1	0.24
SER41-Side-OG	LIG155-Side-O33	0.04
LYS39-Side-NZ	LIG155-Side-O33	0.06
LIG155-Side-O34	TRP116-Main-O	0.04
LIG155-Side-O34	ILE40-Main-O	0.02
HIE96-Side-NE2	LIG155-Side-F19	0.07
LIG155-Side-N25	ASP94-Side-OD1	0.01
LIG155-Side-N25	ASP94-Side-OD2	0.01
TYR95-Side-OH	LIG155-Side-N22	0.02
HIE96-Side-NE2	LIG155-Side-N22	0.68
LIG155-Side-N25	ASN114-Side-OD1	0.13
TYR95-Side-OH	LIG155-Side-O33	0.01
HIE96-Side-NE2	LIG155-Side-O33	0.01
LIG155-Side-O34	GLU86-Side-OE2	0.32
LIG155-Side-O34	GLU86-Side-OE1	0.05
HIE109-Side-NE2	LIG155-Side-F19	0.04
ASN114-Side-ND2	LIG155-Side-N25	0.01
ASN114-Side-ND2	LIG155-Side-N24	0.01
ASN114-Side-ND2	LIG155-Side-O33	0.06
LYS107-Side-NZ	LIG155-Side-N22	0.05
LIG155-Side-N25	GLU86-Side-OE1	0.03

**Table 3:** MM/GBSA and MM/PBSA binding energies for P-Rex1/compound complexes obtained through MD simulation trajectories. The values are shown in kcal/mol.

Energy component	MM/GBSA	MM/PBSA	MM/GBSA	MM/PBSA	MM/GBSA	MM/PBSA
	Top-1	Top-1	Top-2	Top-2	Top-3	Top-3
Δ*G* binding	-30.06	-1.53	-14.35	-1.13	-20.51	-2.87
Δ*G* electrostatic	3.28	3.28	-0.84	-0.84	-1.74	-1.74
Δ*G* bind Van der Waals	-41.3	-41.3	-21.16	-21.16	-22.54	-22.54
Δ*G* bind gas phase	-38.02	-38.02	-22.01	-22.01	-24.28	-24.28
Δ*G* polar solvation	12.87	17.04	9.94	8.33	8.94	28.22
Δ*G* nonpolar solvation	-4.91	-30.2	-2.29	-13.83	-5.17	-6.81
EDISPER energy	-	49.65	-	26.36	-	7.03
Δ*G* solvation	7.96	19.63	7.65	20.87	3.77	21.41

**Table 4:** Computational druglikeness and pharmacokinetics of compounds.

	Property	Compound	
		Top-1	Top-2
Physicochemical properties	Formula	C23H25ClN4O2S	C25H15ClF2N4O2
	Molecular weight	456.99 g/mol	476.86 g/mol
	Num. heavy atoms	31	34
	Num. arom. heavy atoms	16	24
	Fraction Csp3	0.39	0.04
	Num. rotatable bonds	5	4
	Num. H-bond acceptors	5	7
	Num. H-bond donors	0	2
	Molar refractivity	128.62	128.21
	TPSA	97.61 Å^2^	87.47 Å^2^

Lipophilicity	Consensus Log Po/w	4.72	4.78

Water solubility	Water solubility	Moderately soluble	Poorly soluble

Pharmacokinetics	GI absorption	High	High
	BBB permeant	No	No
	P-gp substrate	No	No
	CYP1A2 inhibitor	No	No
	CYP2C19 inhibitor	Yes	No
	CYP2C9 inhibitor	Yes	Yes
	CYP2D6 inhibitor	No	No
	CYP3A4 inhibitor	Yes	No
	Log Kp (skin permeation)	-5.64 cm/s	-5.53 cm/s

Druglikeness	Lipinski	Yes	Yes; 0 violation
	Ghose	Yes	No
	Veber	Yes	Yes
	Egan	Yes	No
	Muegge	Yes	No
	Bioavailability score	0.55	0.56

Medicinal chemistry	PAINS	0 alert	0 alert
	Brenk	0 alert	0 alert
	Synthetic accessibility	4.64	3.80

Toxicity	Hepatotoxicity	N0	Yes
	Skin sensitisation	No	No
	*T. pyriformis* toxicity	No	No
	AMES toxicity	No	No
	Minnow toxicity	No	No
	Carcino mouse	Negative	Negative

Excretion	Total clearance	-0.082	0.075
	Renal OCT2 substrate	No	No

## Data Availability

The original contributions presented in the study can be found in online repositories and also included in the article/supplementary material; further inquiries can be directed to the corresponding authors.

## Supplementary Materials

Supplementary Materials S-Figure 1. Top-1-P-Rex1 complex in TIP3 waterbox. The P-Rex1 is colored by secondary structure elements where the top-1 compound is shown by a yellow ball and stick. The red and white chemical entities represent water molecules surrounding the complex.

## References

[1] Hanahan D., Weinberg R. A. (2011). Hallmarks of cancer: the next generation. *Cell*.

[2] Weber G. F. (2013). Why does cancer therapy lack effective anti-metastasis drugs?. *Cancer Letters*.

[3] Chang J.-W. W., Ding Y., ul Qamar M. T., Shen Y., Gao J., Chen L. L. (2019). A deep learning model based on sparse auto-encoder for prioritizing cancer-related genes and drug target combinations. *Carcinogenesis*.

[4] Khan W., Ashfaq U. A., Aslam S. (2017). Anticancer screening of medicinal plant phytochemicals against cyclin-dependent kinase-2 (CDK2): an in-silico approach. *Advancements in Life Sciences*.

[5] Muneer I., Ahmad S., Naz A. (2021). Discovery of novel inhibitors from medicinal plants for V-domain Ig suppressor of T-cell activation. *Frontiers in Molecular Biosciences*.

[6] Cash J. N., Davis E. M., Tesmer J. J. G. (2016). Structural and biochemical characterization of the catalytic core of the metastatic factor P-Rex1 and its regulation by PtdIns(3,4,5) _P_ _3_. *Structure*.

[7] Suleman M., ul Qamar M. T., Saleem S. (2021). Mutational landscape of Pirin and elucidation of the impact of most detrimental missense variants that accelerate the breast cancer pathways: a computational modelling study. *Frontiers in Molecular Biosciences*.

[8] Riaz M., Ashfaq U. A., Qasim M., Yasmeen E., Ul Qamar M. T., Anwar F. (2017). Screening of medicinal plant phytochemicals as natural antagonists of p53-MDM2 interaction to reactivate p53 functioning. *Anti-Cancer Drugs*.

[9] Berger M. F., Hodis E., Heffernan T. P. (2012). Melanoma genome sequencing reveals frequent *PREX2* mutations. *Nature*.

[10] Lindsay C. R., Lawn S., Campbell A. D. (2011). P-Rex1 is required for efficient melanoblast migration and melanoma metastasis. *Nature Communications*.

[11] Kim E. K., Yun S. J., Ha J. M. (2012). Synergistic induction of cancer cell migration regulated by G*βγ* and phosphatidylinositol 3-kinase. *Experimental & Molecular Medicine*.

[12] Welch H. C. E., Condliffe A. M., Milne L. J. (2005). P-Rex1 regulates neutrophil function. *Current Biology*.

[13] Donald S., Hill K., Lecureuil C. (2004). P-Rex2, a new guanine-nucleotide exchange factor for Rac. *FEBS Letters*.

[14] Welch H. C. E., Coadwell W. J., Ellson C. D. (2002). P-Rex1, a PtdIns(3,4,5)P_3_- and G*βγ*-regulated guanine- nucleotide exchange factor for Rac. *Cell*.

[15] Hill K., Krugmann S., Andrews S. R. (2005). Regulation of P-Rex1 by phosphatidylinositol (3,4,5)-trisphosphate and G*βγ* subunits. *The Journal of Biological Chemistry*.

[16] Rosenfeldt H., Vázquez-Prado J., Gutkind J. S. (2004). P-REX2, a novel PI-3-kinase sensitive Rac exchange factor. *FEBS Letters*.

[17] Li Z., Paik J.-H., Wang Z., Hla T., Wu D. (2005). Role of guanine nucleotide exchange factor P-Rex-2b in sphingosine 1-phosphate-induced Rac1 activation and cell migration in endothelial cells. *Prostaglandins & Other Lipid Mediators*.

[18] Dong X., Mo Z., Bokoch G., Guo C., Li Z., Wu D. (2005). P-Rex1 is a primary Rac2 guanine nucleotide exchange factor in mouse neutrophils. *Current Biology*.

[19] Jaiswal M., Dvorsky R., Ahmadian M. R. (2013). Deciphering the molecular and functional basis of Dbl family proteins:. *The Journal of Biological Chemistry*.

[20] Urano D., Nakata A., Mizuno N., Tago K., Itoh H. (2008). Domain-domain interaction of P-Rex1 is essential for the activation and inhibition by G protein *βγ* subunits and PKA. *Cellular Signalling*.

[21] Lipinski C. A. (2004). Lead- and drug-like compounds: the rule-of-five revolution. *Drug Discovery Today: Technologies*.

[22] Spławiński J., Kuźniar J., Filipiak K., Zieliński W. (2006). Evaluation of drug toxicity in clinical trials. *Science and Engineering Ethics*.

[23] Whitty A. (2011). Growing PAINS in academic drug discovery. *Future Medicinal Chemistry*.

[24] Cheng T., Li Q., Zhou Z., Wang Y., Bryant S. H. (2012). Structure-based virtual screening for drug discovery: a problem-centric review. *The AAPS Journal*.

[25] Karplus M., McCammon J. A. (2002). Molecular dynamics simulations of biomolecules. *Nature Structural Biology*.

[26] ul Qamar M. T., Mirza M. U., Song J.-M., Rao M. J., Zhu X., Chen L.-L. (2021). Probing the structural basis of Citrus phytochrome B using computational modelling and molecular dynamics simulation approaches. *Journal of Molecular Liquids*.

[27] Ahmad F., Albutti A., Tariq M. H., Din G., ul Qamar M. T., Ahmad S. (2022). Discovery of potential antiviral compounds against Hendra virus by targeting its receptor-binding protein (G) using computational approaches. *Molecules*.

[28] Hou T., Wang J., Li Y., Wang W. (2011). Assessing the performance of the MM_PBSA and MM_GBSA methods. 1. The Accuracy.pdf. *Journal of Chemical Information and Modeling*.

[29] Qamar M. T. U., Saba Ismail S. A., Mirza M. U., Abbasi S. W., Ashfaq U. A., Chen L.-L. (2021). Development of a novel multi-epitope vaccine against Crimean-Congo hemorrhagic fever virus: an integrated reverse vaccinology, vaccine informatics and biophysics approach. *Frontiers in Immunology*.

[30] Woods C. J., Malaisree M., Michel J., Long B., McIntosh-Smith S., Mulholland A. J. (2014). Rapid decomposition and visualisation of protein-ligand binding free energies by residue and by water. *Faraday Discussions*.

[31] Woods C. J., Malaisree M., Hannongbua S., Mulholland A. J. (2011). A water-swap reaction coordinate for the calculation of absolute protein-ligand binding free energies. *The Journal of Chemical Physics*.

[32] Abro A., Azam S. S. (2016). Binding free energy based analysis of arsenic (+ 3 oxidation state) methyltransferase with *S*-adenosylmethionine. *Journal of Molecular Liquids*.

[33] Muneer I., Ul Qamar M. T., Tusleem K., Abdul Rauf S., Hussain H. M. J., Siddiqi A. R. (2019). Discovery of selective inhibitors for cyclic AMP response element-binding protein: a combined ligand and structure-based resources pipeline. *Anti-Cancer Drugs*.

[34] Pires D. E. V., Blundell T. L., Ascher D. B. (2015). pkCSM: predicting small-molecule pharmacokinetic and toxicity properties using graph-based signatures. *Journal of Medicinal Chemistry*.

[35] Daina A., Michielin O., Zoete V. (2017). SwissADME: a free web tool to evaluate pharmacokinetics, drug-likeness and medicinal chemistry friendliness of small molecules. *Scientific Reports*.

[36] Islam S., Hosen M. A., Ahmad S. (2022). Synthesis, antimicrobial, anticancer activities, PASS prediction, molecular docking, molecular dynamics and pharmacokinetic studies of designed methyl *α*-D-glucopyranoside esters. *Journal of Molecular Structure*.

[37] Lyu C., Chen T., Qiang B. (2021). CMNPD: a comprehensive marine natural products database towards facilitating drug discovery from the ocean. *Nucleic Acids Research*.

[38] Mumtaz A., Ashfaq U. A., ul Qamar M. T. (2017). MPD3: a useful medicinal plants database for drug designing. *Natural Product Research*.

[39] Maia E. H. B., Assis L. C., de Oliveira T. A., da Silva A. M., Taranto A. G. (2020). Structure-based virtual screening: from classical to artificial intelligence. *Frontiers in Chemistry*.

[40] Pettersen E. F., Goddard T. D., Huang C. C. (2004). UCSF Chimera—a visualization system for exploratory research and analysis. *Journal of Computational Chemistry*.

[41] Abbasi S., Raza S., Azam S. S., Liedl K. R., Fuchs J. E. (2016). Interaction mechanisms of a melatonergic inhibitor in the melatonin synthesis pathway. *Journal of Molecular Liquids*.

[42] Hooft R. W. W., Sander C., Vriend G. (1997). Objectively judging the quality of a protein structure from a Ramachandran plot. *Bioinformatics*.

[43] Jones G., Willett P., Glen R. C., Leach A. R., Taylor R. (1997). Development and validation of a genetic algorithm for flexible docking^1^. *Journal of Molecular Biology*.

[44] Dallakyan S., Olson A. J. (2015). Small-molecule library screening by docking with PyRx. *Chemical Biology*.

[45] Cash J. N., Chandan N. R., Hsu A. Y. (2020). Discovery of small molecules that target the Phosphatidylinositol (3,4,5) Trisphosphate (PIP3)-Dependent Rac Exchanger 1 (P-Rex1) PIP3-binding site and inhibit P-Rex1-dependent functions in neutrophils. *Molecular Pharmacology*.

[46] Alamri M. A., ul Qamar M. T., Afzal O., Alabbas A. B., Riadi Y., Alqahtani S. M. (2021). Discovery of anti-MERS-CoV small covalent inhibitors through pharmacophore modeling, covalent docking and molecular dynamics simulation. *Journal of Molecular Liquids*.

[47] ul Qamar M. T., Ahmad S., Khan A. (2021). Structural probing of HapR to identify potent phytochemicals to control *Vibrio cholera* through integrated computational approaches. *Computers in Biology and Medicine*.

[48] Schepers B., Gohlke H. (2021). Erratum:“AMBER-DYES in AMBER: Implementation of fluorophore and linker parameters into AmberTools”[J. Chem. Phys. 152, 221103 (2020)]. *The Journal of Chemical Physics*.

[49] Khalid R. R., ul Qamar M. T., Maryam A. (2018). Comparative studies of the dynamics effects of BAY60-2770 and BAY58-2667 binding with human and bacterial H-NOX domains. *Molecules: A Journal of Synthetic Chemistry and Natural Product Chemistry*.

[50] Schafmeister C., Ross W. S., Romanovski V. (1995). *The Leap Module of AMBER*.

[51] Case D. A., Babin V., Berryman J. T. (2014). The FF14SB force field. *Amber*.

[52] Dickson C. J., Rosso L., Betz R. M., Walker R. C., Gould I. R. (2012). GAFFlipid: a general amber force field for the accurate molecular dynamics simulation of phospholipid. *Soft Matter*.

[53] Petersen H. G. (1995). Accuracy and efficiency of the particle mesh Ewald method. *The Journal of Chemical Physics*.

[54] Kräutler V., Van Gunsteren W. F., Hünenberger P. H. (2001). A fast SHAKE algorithm to solve distance constraint equations for small molecules in molecular dynamics simulations. *Journal of Computational Chemistry*.

[55] Izaguirre J. A., Catarello D. P., Wozniak J. M., Skeel R. D. (2001). Langevin stabilization of molecular dynamics. *The Journal of Chemical Physics*.

[56] Roe D. R., Cheatham T. E. (2013). PTRAJ and CPPTRAJ: software for processing and analysis of molecular dynamics trajectory data. *Journal of Chemical Theory and Computation*.

[57] Haq F. U., Abro A., Raza S., Liedl K. R., Azam S. S. (2017). Molecular dynamics simulation studies of novel *β*-lactamase inhibitor. *Journal of Molecular Graphics & Modelling*.

[58] Maiorov V. N., Crippen G. M. (1994). Significance of root-mean-square deviation in comparing three-dimensional structures of globular proteins.

[59] Lobanov M. Y., Bogatyreva N. S., Galzitskaya O. V. (2008). Radius of gyration as an indicator of protein structure compactness. *Molecular Biology*.

[60] Iqbal S., Shamim A., Azam S. S., Wadood A. (2016). Identification of potent inhibitors for chromodomain-helicase- DNA-binding protein 1-like through molecular docking studies. *Medicinal Chemistry Research*.

[61] Wade R. C., Goodford P. J. (1989). The role of hydrogen-bonds in drug binding. *Progress in Clinical and Biological Research*.

[62] Donohue J. (1954). Radial distribution functions of some structures of the polypeptide chain. *Proceedings of the National Academy of Sciences*.

[63] Raza S., Azam S. S. (2018). AFD: an application for bi-molecular interaction using axial frequency distribution. *Journal of Molecular Modeling*.

[64] Ahmad F., Azam S. S. (2020). Role of ring positioning and preferential occupation of ligand obtained through molecular dynamics simulation of peptidoglycan associated lipoprotein (Pal). *Journal of Molecular Graphics and Modelling*.

[65] Altharawi A., Ahmad S., Alamri M. A., Qamar M. T. u. (2021). Structural insight into the binding pattern and interaction mechanism of chemotherapeutic agents with sorcin by docking and molecular dynamic simulation. *Colloids Surfaces B Biointerfaces*.

[66] Miller B. R., McGee T. D., Swails J. M., Homeyer N., Gohlke H., Roitberg A. E. (2012). MMPBSA.py: an efficient program for end-state free energy calculations. *Journal of Chemical Theory and Computation*.

[67] Duan L., Liu X., Zhang J. Z. H. (2016). Interaction entropy: a new paradigm for highly efficient and reliable computation of protein--ligand binding free energy. *Journal of the American Chemical Society*.

[68] Moreira I. S., Fernandes P. A., Ramos M. J. (2007). Computational alanine scanning mutagenesis—an improved methodological approach. *Journal of Computational Chemistry*.

[69] Lee S. K., Lee I. H., Kim H. J., Chang G. S., Chung J. E., No K. T. (2003). The PreADME approach: web-based program for rapid prediction of physico-chemical, drug absorption and drug-like properties. *EuroQSAR 2002 Designing Drugs and Crop Protectants: Processes, Problems and Solutions*.

[70] De Boer A. G., Breimer D. D. (1994). The blood-brain barrier: clinical implications for drug delivery to the brain. *Journal of the Royal College of Physicians of London*.

[71] Stead A. G., Hasselblad V., Creason J. P., Claxton L. (1981). Modeling the Ames test. *Mutation Research/Environmental Mutagenesis and Related Subjects*.

[72] Lombardo F., Desai P. V., Arimoto R. (2017). In SilicoAbsorption, distribution, metabolism, excretion, and pharmacokinetics (ADME-PK): utility and best practices. An industry perspective from the international consortium for innovation through quality in pharmaceutical Development. *Journal of Medicinal Chemistry*.

[73] Ferreira L. G., Dos Santos R. N., Oliva G., Andricopulo A. D. (2015). Molecular docking and structure-based drug design strategies. *Molecules*.

[74] Meng X.-Y., Zhang H.-X., Mezei M., Cui M. (2011). Molecular docking: a powerful approach for structure-based drug discovery. *Current Computer-Aided Drug Design*.

[75] Shivanika C. (2022). Molecular docking, validation, dynamics simulations, and pharmacokinetic prediction of natural compounds against the SARS-CoV-2 main-protease. *Journal of Biomolecular Structure and Dynamics*.

[76] Elfiky A. A., Azzam E. B. (2021). Novel guanosine derivatives against MERS CoV polymerase: an in silico perspective. *Journal of Biomolecular Structure and Dynamics*.

[77] Elmezayen A. D. (2021). Drug repurposing for coronavirus (COVID-19): in silico screening of known drugs against coronavirus 3CL hydrolase and protease enzymes. *Journal of Biomolecular Structure and Dynamics*.

[78] Genheden S., Ryde U. (2015). The MM/PBSA and MM/GBSA methods to estimate ligand-binding affinities. *Expert Opinion on Drug Discovery*.

[79] Ghose A. K., Viswanadhan V. N., Wendoloski J. J. (1999). A knowledge-based approach in designing combinatorial or medicinal chemistry libraries for drug discovery. 1. A qualitative and quantitative characterization of known drug databases. *Journal of Combinatorial Chemistry*.

[80] Veber D. F., Johnson S. R., Cheng H. Y., Smith B. R., Ward K. W., Kopple K. D. (2002). Molecular properties that influence the oral bioavailability of drug candidates. *Journal of Medicinal Chemistry*.

[81] Egan W. J., Merz K. M., Baldwin J. J. (2000). Prediction of drug absorption using multivariate statistics. *Journal of Medicinal Chemistry*.

[82] Muegge I., Heald S. L., Brittelli D. (2001). Simple selection criteria for drug-like chemical matter. *Journal of Medicinal Chemistry*.

[83] Zhang Y., Benet L. Z. (2001). The gut as a barrier to drug absorption. *Clinical Pharmacokinetics*.

[84] Wang Z., Chen Y., Liang H., Bender A., Glen R. C., Yan A. (2011). P-glycoprotein substrate models using support vector machines based on a comprehensive data set. *Journal of Chemical Information and Modeling*.

