# Supplementary material for: Structural Dynamics of P-Rex1 Complexed with Natural Leads Establishes the Protein as an Attractive Target for Therapeutics to Suppress Cancer Metastasis
Source: Biomed Res Int. 2023 Dec 7;2023:3882081. doi: 10.1155/2023/3882081 (PMC10721353; doi:10.1155/2023/3882081)
Supplement: Supplementary Materials — S-Figure 1. Top-1-P-Rex1 complex in TIP3 waterbox. The P-Rex1 is colored by secondary structure elements where the top-1 compound is shown by a yellow ball and stick. The red and white chemical entities represent water molecules surrounding the complex. [file 3882081.f1.docx]

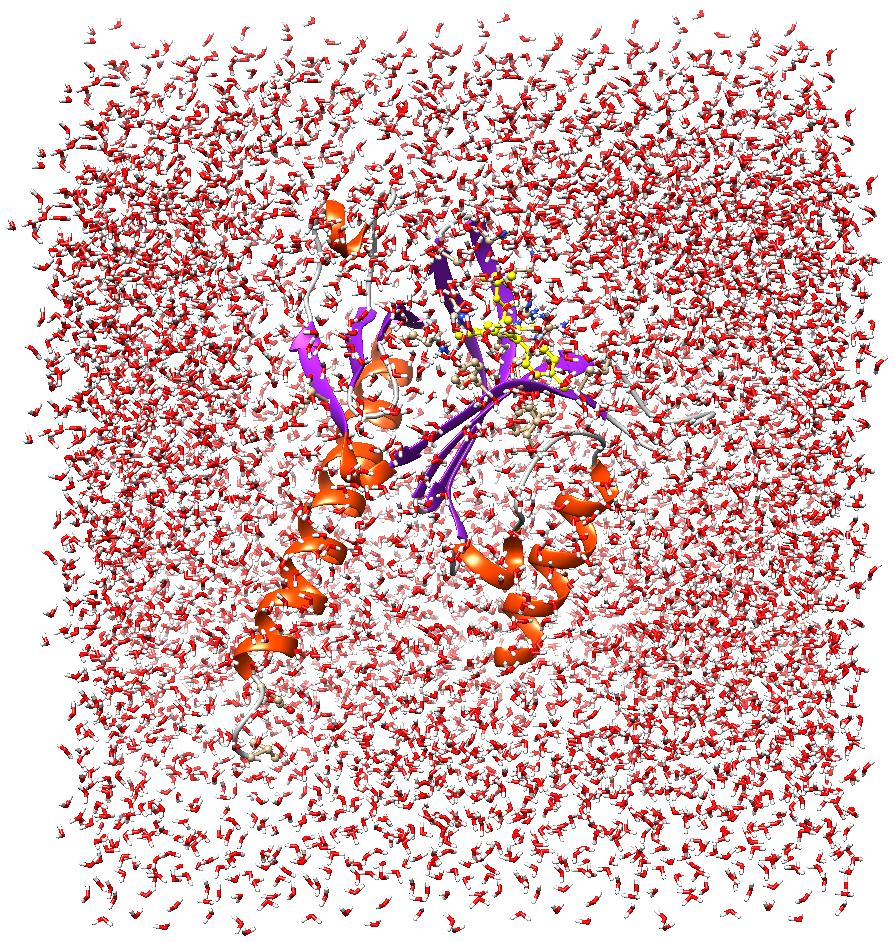


**S-Fig.1.** Top-1- P-Rex1 complex in TIP3 waterbox. The P-Rex1 is colored by secondary structure elements where Top-1 compound is shown by yellow ball and stick. The red and white chemical entities represent water molecules surrounding the complex.
